# Mortality markers in nosocomial *Klebsiella pneumoniae* bloodstream infection

**DOI:** 10.1186/s40064-016-3580-8

**Published:** 2016-10-28

**Authors:** Bulent Durdu, Ismail Necati Hakyemez, Sibel Bolukcu, Gulay Okay, Bilge Gultepe, Turan Aslan

**Affiliations:** 1 Department of Infectious Diseases and Clinical Microbiology, Faculty of Medicine, Bezmialem Vakif University, Adnan Menderes Boulevard, Fatih, 34093 Istanbul, Turkey; 2 Department of Microbiology and Clinical Microbiology, Faculty of Medicine, Bezmialem Vakif University, Istanbul, Turkey

## Abstract

**Purpose:**

*Klebsiella pneumoniae* is the most common endogen agent for nosocomial infections. In this study, mortality markers were investigated in patients with nosocomial *K. pneumoniae* blood stream infection (NKp BSI).

**Methods:**

The characteristics of patients >16 years who had NKp BSI diagnosis by daily active surveillance between January 2012 and January 2016 were retrospectively evaluated. Patients who died until 28th day of the clinical follow up and those who survived until this time were statistically compared in terms of various risk factors.

**Results:**

One hundred ninety patients were included into the study. Mortality rate was 47.9%, carbapenem resistance was 43.2%. Statistical analysis have shown that in presence of post-NKp BSI sepsis, septic shock, following in intensive care unit (ICU), meropenem resistance, kidney failure, NKp BSI secondary to pneumonia, use of invasive instruments such as central venous catheter (CVC), urinary catheter (UC) and mechanical ventilator (MV), colostomy, transfusion and hemodialysis mortality was significantly higher. In patients admitted into the hospital for neurological disorders, pancreaticobiliary tract (PBT) diseases and patients who have undergone endoscopic retrograde cholangiopancreatography (ERCP) and patients in whom NKp BSI secondary to PBT infection mortality rate was lower.

**Conclusions:**

Sepsis, septic shock, clinical conditions requiring ICU treatment and meropenem resistance increase mortality rates in NKp BSI significantly. Mortality was higher also in patients with NKp BSI secondary to pneumonia, in kidney failure and when invasive instruments were used. On the other hand, in patients who were admitted to the hospital for neurological disorders and PBT diseases mortality rate was lower.

## Background

Currently, due to increase in surgical procedures, intensive use of invasive instruments, immunosuppressive therapy and other similar practices incidence of health care associated infections (HAI) and its significance have increased worldwide (Kaiser et al. 2013; Kontopidou et al. 2014; Alicino et al. 2015). BSI, either HAI or community-acquired are among the most severe life–threatening infections (Alicino et al. 2015). Enterobacteriaceae are among the most important HAI source (Tumbarello et al. 2012). Within enterobacteriaceae family *K. pneumoniae* is the most common microorganism isolated as a source of infection in HAI (Tumbarello et al. 2012; Ben-David et al. 2012; Pau et al. 2015; Vardakas et al. 2015).

In the last decade, multi drug resistance (MDR) and the increase in incidence of infections due to Gram negative bacilli producing extended spectrum β lactamase (ESBL) led to the intense use of carbapenem. Because of this, *Klebsiella pneumoniae* (CRKp) resistant to carbapenem was first reported in 1996 and since then the prevalence of carbapenem resistance have reached to life threatening endemic dimensions in various regions particularly among populations exposed to health care (Ben-David et al. 2012; Gomez-Simmonds et al. 2015). Behindhand discovery of novel effective antibiotics relative to increasing bacterial resistance rates pose difficulty in treating infections and increases mortality rates (Chen et al. 2012). In studies investigating NKp BSI outcomes crude mortality rate was reported at a high range between 23 and 69% (Kontopidou et al. 2014; Tumbarello et al. 2012; Ben-David et al. 2012; Pau et al. 2015; Vardakas et al. 2015; van Duin et al. 2014; Hoxha et al. 2016; Ulu et al. 2015; Kang et al. 2006; Daikos et al. 2014; Correa et al. 2013). Structure of nosocomial infections is dynamic and complex and usually includes multiple variables negatively effecting clinical outcome. Furthermore, in the initial period of this infection there are additional uncertainties in terms of type of the causative microorganisms and antibiotic susceptibility. Thus, empirical antibiotic therapy is the most critical step in the initial period for combating with the infection. Minimum 2–3 days needed to assess the effectiveness of the antibiotic and to resolve uncertainties about the type of microorganisms further increases the importance of this critical step.

The increasing incidence of NKp BSI, limited therapeutic options and uncertainties in the initial period of the infection leading to higher mortality rates created a need for studies focusing on detecting risk factors having an impact on survival and investigating the effect of empirical therapy.

Worldwide there are various studies on this subject; however, the clinical studies demonstrating the current condition in our country are scarce. Thus, in our hospital we aimed to detect risk factors effecting mortality rate of patients with NKp BSI and to investigate the effect of empirical antibiotic therapy on clinical outcome.

## Methods

Patients aged >16 years, who were hospitalized between January 1st, 2012 and January 1st, 2016 with clinical features of BSI, with a period of time >48 h passing after hospitalization, with *K. pneumoniae* growth in at least one hemoculture were evaluated retrospectively. The patient was excluded if BSI was polybacterial, if the patient could not be followed-up or his/her medical data could not be accessed. If *K. pneumoniae* had caused more than one BSI in a patient, the first episode was evaluated.

This study was done at Bezmialem Vakif University Medical School Hospital with 550 beds (Istanbul, Turkey). Daily BSI surveillance was done based on patient and laboratory by infectious diseases specialists and infection control nurses in our hospital. Patient data were recorded manually on surveillance forms prepared by the Ministry of Health.

The demographic data, cause of hospitalization, co-morbidities, risk factors, possible sources of infection, sepsis, development of septic shock, type of surgical intervention, antimicrobial susceptibility and information on initial empirical antibiotic use were obtained from surveillance forms. The dates of hemocultures were obtained from the electronic data system on which patient data were recorded.

Sepsis and septic shock were diagnosed according to guidelines (Vincent et al. 2013). Those patients in whom monitorization was considered to be indicated in the ICU in the first 48 h after obtaining culture samples or those whose monitorization at the ICU were decided to be continued in the first 48 h after diagnosis of BSI were considered as requirement for ICU.

HAI was diagnosed during daily active surveillance according to Centers for Disease Control and Prevention criteria and recorded (Sievert et al. 2013). Possible risk factors were selected in light of the medical literature. ERCP and blood/blood product transfusion were considered as a risk factor if done in the last 5 days before BSI and chemotherapy (CT) if done in the last 3 weeks. Surgical drain, MV, CVC, UC, total parenteral nutrition (TPN), hemodialysis (HD) and colostomy were considered relevant if only their use were initiated before development of BSI and were still continuing at the time of BSI development. Surgical interventions that were done in the month before BSI were classified according to systems and were recorded.

The result of antimicrobial susceptibility was considered as resistant to carbapenem (CpR) if resistance against meropenem or imipenem was present, and considered as susceptible to carbapenem if sensitive to both (CpS).

### Microbiology

Hemoculture tubes (Becton–Dickinson, USA) sent to the microbiology laboratory of our hospital were put in the BACTEC FX (Becton–Dickinson, USA) device, programmed for an incubation period of 5 days. In samples with positive signals during this period, first a gram staining were done from the bottle, after which incubations were done in blood agar (Salubris, Turkey), EMB agar (Salubris, Turkey) and chocolate agar (Salubris, Turkey) at 37 °C for 18–24 h. The bacteria that had grown were defined with conventional methods and automated systems (VITEK^®^ 2 Compact (BioMérieux, France), and antimicrobial susceptibility results obtained with VITEK-2 were evaluated according to Clinical and Laboratory Standards Institute (CLSI) (2013) criteria. The strains identified with Vitec^®^ 2 Compact (BioMérieux, France) device were also examined with Vitec^®^ MS (MALDI-TOF) (BioMérieux, France) device for identification and confirmation and were found to be *K. pneumoniae*. In this study, *K. pneumoniae* strains that were isolated from the same clinical samples of the same patient, showing similar antimicrobial susceptibility patterns were not evaluated. The MIC values found for colistin were also evaluated according to breakpoint test values of The European Committee on Antimicrobial Susceptibility Testing (EUCAST).

### Statistical analysis

Data was analyzed using SPSS 13.0 (Chicago, IL, USA). Continuous variables were described as mean ± standard deviation and range. Percentage values were described without decimal. To evaluate risk factors associated with mortality, Pearson χ^2^ test, or Fisher’s exact test for categorical variables and unpaired Student’s t test for continuous variables were performed. Bivariate logistic regression analysis was conducted to obtain unadjusted hazard ratios and revealed as (Hazard ratio (HR); 95% Confidence interval; P value) NKp BSI in comparison of both survivor and non-survivor groups. Risk factors that reached statistical significance (P < 0.05) using a forward selection process remained in the model. All tests were based on two-tailed tests and p < 0.05 were considered as significant.

## Results

Two hundred ant two patients were included in this study. Seven patients were excluded as they were referred to other centers due to various causes and could not be followed-up, and five patients were excluded due to isolation of multiple agents. Findings, p values, hazards ratios and confidence intervals are summarized at Tables 1 and 2. One hundred and fourteen patients (59.8%) were males and 76 (40.2%) were females. Age distribution was between 20 and 93 years, and mean age was 62.71 (±16.4) years. The mean age of males was 60.89 (±16.18) years, and 65.42 (±16.46) years in females. The sections that the patients were hospitalized were as follows; 63.2% at the ICU, 21.6% at internal medicine wards, 15.2% in surgical wards. The number of patients who died until the 28th day was 91 (47.9%), and the number of those surviving was 99 (52.1%). When mortality rates were evaluated according to sections, 63.3% died at the ICU, 29.3% died at internal medicine sections and 10.3% died at surgical wards. The mean age of patients that died until the 28th day was 64.91 (±15.54) years and the mean age of those surviving was 60.67 (±14.94) years. The range of hospital stay before development of BSI was 3–122 days, with a mean of 27.82 (±26.85) days in those who died and 20.65 (±19.57) days in hose who survived. The time interval after diagnosis of BSI till discharge was between 0 and 193 days, with a mean duration of 12.81 (±10.8) days in who had died, and 33.15 (±28.62) days in those who survived (Table 1).

**Table 1:** Clinical variables and relationship with day 28th mortality

Variable	Died (91)		Living (99)		P value	HR (CI)
	N	%	N	%		
Demographic data						
Mean age	64.91 ± 15.54		60.67 ± 14.94		0.074	
Hospital stay before BSI	27.82 ± 26.85		20.65 ± 19.57		0.085	
Hospital stay after BSI	12.81 ± 10.8		33.15 ± 28.62		*0.0005*	
Male sex	56	61.5	58	58.6	0.678	
Infection complication						
Sepsis	91	100	76	76.8	*0.0005*	*2.197 (1.86–2.59)*
Septic shock	81	89	24	24.2	*0.0005*	*25.31 (11.35–56.43)*
Need for ICU after infection	91	100	66	66.7	*0.0005*	*2.37 (1.98–2.85 43)*
BSI Source						
CLABSI	25	27.5	31	31.3		
Pneumonia	29	31.9	15	15.2	*0.006*	*2.61 (1.29–5.29)*
Pancreaticobiliary tract	8	8.8	27	27.3	*0.001*	*0.25 (0.11–0.6)*
Primer BSI	12	13.2	8	8.1	0.251	
SSI	10	11	9	9.1	0.810	
UTI	6	6.6	7	7.1	0.896	
YDE	2	2.2	2	2	1.00	
Indication of admittance into hospital						
Malignancy	28	30.8	36	36.4	0.415	
Acute respiratory failure	26	28.6	22	22.2	0.314	
Pancreaticobiliary tract infection	9	9.9	23	23.2	*0.013*	*0.36 (0.15–0.83)*
Neurological disorder	9	9.9	23	23.2	*0.019*	*0.15 (0.36–0.6)*
Pneumonia	17	18.7	13	13.1	0.295	
Acute abdomen	11	12.1	10	10.1	0.663	
Trauma	8	8.8	10	10.1	0.758	
Acute renal failure	13	14.3	5	5.1	*0.028*	*3.13 (1.07–9.17)*
Cardiac disease	11	12.1	6	6.1	0.144	
Sepsis	8	8.8	6	6.1	0.422	
UTI	4	4.4	4	4	0.903	
Chronic live disease	4	4.4	3	3	0.618	
Co–morbid disease						
Hypertension	45	49.5	50	50.5	0.885	
Malignancy	34	37.4	43	43.4	0.394	
Diabetes mellitus	27	29.7	25	25.3	0.495	
Neurological disorder	21	23.1	27	27.3	0.506	
Coronary artery disease	14	15.4	26	26.3	0.064	
Chronic liver failure	27	29.7	11	11.1	*0.001*	*3.37 (1.56–7.3)*
Cardiac insufficiency	15	16.5	15	15.2	0.801	
Chronic obstructive pulmonary disease	10	11	14	14.1	0.512	
Hepatic failure	5	5.5	6	6.1	0.867	
Risk factor						
Urinary catheter	76	83.5	66	66.7	*0.007*	*2.53 (1.26–5.07)*
CVC	71	78	63	63.6	*0.029*	*2.02 (1.06–3.86)*
Mechanic ventilation	69	75.8	56	56.6	*0.005*	*2.4 (1.29–4.49)*
Blood and blood product transfusion	51	56	41	41.4	*0.043*	*1.8 (1.01–3.2)*
Drainage	38	41.8	34	34.3	0.293	
Total parenteral nutrition	31	34.1	28	28.3	0.390	
ERCP	9	9.9	25	25.3	*0.005*	*0.32 (0.14–0.74)*
Hemodialysis	20	22	9	9.1	*0.013*	*2.81 (1.2–6.56)*
Chemotherapy	16	17.6	9	9.1	0.082	
Colostomy	12	13.2	2	2	*0.002*	*7.36 (1.6–33.89)*
Previous surgical procedure						
Gastrointestinal system	14	15.4	14	14.1	0.809	
Musculo-skeletal system	10	11	10	10.1	0.842	
Central nervous system	7	7.7	8	8.1	0.921	
Genitourinary system	4	4.4	9	9.1	0.256	
Bile ducts, liver and pancreas	8	8.8	4	4	0.236	
Thorax surgery	4	4.4	0	0	1.00	
Cardiovascular surgery	1	1.1	1	1	0.051	
Operation	41	45.1	42	42.4	0.715	
Appropriate empirical antibiotic therapy	49	53.8	57	57.6	0.662	

*BSI* bloodstream infection; *ICU* intensive care units; *HR* hazard ratio; *CI* confidence interval; *SSI* surgical site infection; *UTI* urinary tract infection; *SSTI* skin and soft tissue infection; *CLABSI* central line-associated bloodstream infection; *CVC* central venous catheter; *ERCP* endoscopic retrograde cholangiopancreatography

**Table 2:** Relationship between antibiotic resistance and mortality

Antibiotic	Died (91)		Living (99)		P value	HR (CI)
	Susceptible		Susceptible			
	N	%	N	%		
Colistin	91	100.0	99	100.0	1.00	
Tigecycline	71	78.0	75	75.8	0.734	
Trimethoprim-sulfametaxazol	59	64.8	56	56.6	0.299	
Meropenem	46	50.5	65	65.7	*0.035*	*0.53 (0.29–0.95)*
Imipenem	46	50.5	63	63.6	0.068	
Meropenem or imipenem	46	50.5	63	63.6	0.067	
Gentamicin	44	48.4	57	57.6	0.245	
Amikacin	40	44.0	48	48.5	0.532	
Levofloxacin	28	30.8	32	32.3	0.876	
Ciprofloxacin	25	27.5	32	32.3	0.466	
Tetracycline	20	22.0	31	31.3	0.190	
Piperacillin-tazobactam	21	23.1	28	28.3	0.412	
Cefoperazone-sulbactam	18	19.8	25	25.3	0.391	
Ceftazidime	8	8.8	12	12.1	0.488	
Cefepime	7	7.7	11	11.1	0.466	
Ampicillin-sulbactam	7	7.7	7	7.1	1.00	

The number of patients in whom sepsis developed was 167 (87.9%), and the number of patients with septic shock was 105 (55.3%). Mortality was significantly higher in those in whom sepsis and septic shock had developed. Mortality rates at the 28th day of patients for whom ICU care was indicated in the first 24 h after BSI or in those whose requirement for ICU care continued were significantly higher (Table 1).

Rates of UC, CVC and MV use were 75.1, 70.9 and 66.1%, respectively. Mortality rates were significantly higher in those in whom UC, CVC and MV were used. Mortality rates were significantly higher in patients in whom hemodialysis, colostomy and blood/blood product transfusions were administered, while mortality was lower in patients in whom ERCP was done (Table 1).

The most frequent cause was central line associated BSI (CLABSI) (29.5%), and the second most frequent cause was pneumonia (22.6%) among primary infection sources that cause NKp BSI. Other possible causes include PBT infections (18.4%), primary BSI (10.5%), surgical area infection (SAI) (10%), urinary tract infections (UTI) (6.8%) and soft tissue infections (STI) (2.1%). All of the patients in whom possible BSI source was pneumonia were those that received MV support in the ICU and mortality was significantly high in this group. On the other hand, mortality was lower in patients in whom the possible source was PBT infections. A significant difference was not observed in others (Table 1).

Among diseases that required hospitalization, most prevalent was malignancy (33.7%), followed by acute respiratory failure (25.3%). Other disorders included PBT infections (16.8%), neurological disorders (16.8%), pneumonia (15.8%), acute abdominal pain (11.1%), trauma (9.5%), acute renal failure (ARF) (9.5%), cardiac disorders (8.9%), sepsis (7.4%), UTI (4.2%) and chronic hepatic diseases (3.7%). Mortality was high in patients in whom the cause of hospitalization was ARF, while it was lower in those with PBT infection and neurological disorders. Mortality on the 28th day was high only in the presence of CRF among co-morbidities (Table 1).

43.7% of the patients had undergone surgical intervention before BSI. Types of surgery included gastrointestinal system (GIS) (14.7%), musculoskeletal system (10.5%), central nervous system (CNS) (7.9%), genitourinary system (GUS) (6.8%), PBT and hepatic surgery (6.3%), thoracic surgery (2.1%) and cardiovascular surgery (CVS) (1.1%), in descending order of frequency. A significant association between mortality on the 28th day was not observed in patients who had or had not undergone surgery before BSI. Also, a significant association between type of surgery and mortality on the 28th day was not observed (Table 1).

Resistance rates of 190 *K. pneumoniae* strains according to CLSI breakpoint values were as follows: tigecycline (TGC) 23.2%, trimethoprim-sulfamethoxazole (TMP-SXT) (39.5%), meropenem (MEM) 41.6%, gentamicin (GM) (46.8%), amikacin (AK) (53.7%), levofloxacin (LEV) (68.4%), ciprofloxacin (CIP) (70.0%), tetracyclin (TE) (73.2%), piperacillin-tazobactam (TZP) (74.2%), cefoperazone-sulbactam (CES) (77.4%), ceftazidim (CAZ) (89.5%), cefepime (FEP) (90.5%), sulbactam-ampicillin (SAM) (92.6%). Although breakpoint values suggested by VITEC II for colistin (CS) was not approved by the US Food and Drug Administration (FDA), 10% resistance was observed, while resistance was not found with EUCAST breakpoint values. When the association of antimicrobial resistance and mortality was examined, mortality was found to be significantly increased in the presence of MEM resistance, while significant changes were not observed in the presence of resistance to other antimicrobials (Table 2). CR was detected in 82 patients (43.2%). Mortality rate was 56.1% in the presence of CR, and 41.7% in the presence CS.

The rate of initiation of appropriate empirical antimicrobial treatment was 55.2% in general study population, while 53.8% in patients who had died and 57.6% in those surviving. A statistically significant difference was not observed between mortality rates of patients who received or did not receive appropriate empirical therapy (Table 1).

## Discussion

In our study mortality rate at the 28th day was high with 47.9%. In the literature, reported mortality rate is within the range 23–69% and the highest mortality rates were observed in patients admitted to ICU and in BSI cases where CRKp is the culprit agent (Kontopidou et al. 2014; Tumbarello et al. 2012; Ben-David et al. 2012; Vardakas et al. 2015; van Duin et al. 2014; Hoxha et al. 2016; Ulu et al. 2015; Kang et al. 2006; Daikos et al. 2014; Correa et al. 2013). We concluded that mortality rate was high in our patients, since most of them were admitted to ICU. The studies on this subject are scarce in our country. In a 2012 study investigating *K. pneumoniae* infections in ICUs a similar mortality rate with 48% was reported (Ulu et al. 2015). These high rates have shown that NKp BSI is a global issue requiring an urgent answer.

Sepsis and septic shock was found to be associated with high mortality. In a Korean study septic shock was found to be an independent risk factor in community acquired infections and NKp BSI (Kang et al. 2006). In studies carried out in USA, Greece and Italy mortality was found to be higher in patients developing septic shock after CRKp infection (Tumbarello et al. 2012; Vardakas et al. 2015; Daikos et al. 2014; Capone et al. 2013). Septic shock is a grave clinical condition leading to failure in maintaining vital functions and loss of function. Monitorization of these patients is usually maintained at ICU under intense medical therapy and by using invasive instruments. Thus, these patients are exposed to adverse effects of medical therapy and under risk of complications due to the use of invasive instruments. Furthermore, extreme age and concomitant diseases paving the way for development of sepsis and septic shock make the clinical picture more severe. Unsurprisingly, these negative factors altogether lead to high mortality. Getting care at ICU has been determined as an independent risk factor related with high mortality rate. In a US study ICU was found to be associated with high mortality (Gomez-Simmonds et al. 2015). In a review mortality rate was reported to be higher in patients developing BSI during their stay at ICU (Petrosillo et al. 2013).

In our study, mortality was significantly higher in patients having NKp BSI secondary to pneumonia. Studies reported from USA, Italy, Hong Kong and Korea also include data supporting our results (Pau et al. 2015; Kang et al. 2006; Capone et al. 2013; Qureshi et al. 2012). Qureshi et al. have assumed that high mortality in BSI secondary to pneumonia was due to the higher severity of underlying disease and relative difficulty in controlling the source of infection in pneumonia compared with other sources (Qureshi et al. 2012). In a review use of MV was emphasized as a factor facilitating colonization with resistant *K. pneumoniae* strains and development of infection (Petrosillo et al. 2013). Also, in all of the patients with BSI secondary to pneumonia use of MV in ICU was the case and that may have lead to inadequate control of infection in these patients. Thus, in patients getting MV support at ICU rigorous attention is mandatory to prevent pneumonia in these patients and infection control measures should also be checked. In case pneumonia has occurred, efforts for prevention of more severe infections such as BSI should be spent by way of early, appropriate treatment.

Survival was found to be higher in patients whom probable BSI source is PBT infection. In the reviewed literature there was only one study investigating the relationship between PBT infection and Kp BSI. In this Korean study, survival rate was found to be significantly high in patients having community-acquired Kp BSI or NKp BSI with PBT infection as the probable source of infection. Observed characteristics of patients with PBT infection were less co-morbidity compared to general population, less exposure to invasive instrument use, shorter hospitalization and lower rate of care at ICU. These were considered as the factors leading to higher survival rates. In line with this, survival rate was high also in patients who have undergone ERCP. Obstructions within the PBT may facilitate development of infection. It’s considered that easy passage from the PBT after ERCP performed with minimal invasiveness and use of prophylactic antibiotics may have facilitated infection control and thereby may have increased survival rate.

In patients hospitalized for ARF or in those having CRF as co-morbid disease but hospitalized for another disorder and in patients getting hemodialysis as supportive therapy mortality rate at Day 28 was found to be significantly higher. In various studies presence of significant correlation with renal failure and mortality was emphasized (Vardakas et al. 2015; Qureshi et al. 2012; Corcione et al. 2014; Hussein et al. 2013; Dubrovskaya et al. 2013). In a study performed in USA use of non-optimal doses of nephrotoxic antibiotics such as CS was blamed in the relationship between renal failure and high mortality (Dubrovskaya et al. 2013). The probable requirement of invasive supportive therapies such as hemodialysis which are risk factors for HAI in renal failure, immunosuppressive effects of CRF and inability to use optimal dose of antibiotic eliminated via kidneys may cause high mortality in presence of RF.

Urinary catheter, CVC and MV use was found to be associated with increasing mortality. In various studies reported mortality rates were high in patients exposed to use of these types of invasive instruments (Ben-David et al. 2012; Gomez-Simmonds et al. 2015; Kang et al. 2006; Petrosillo et al. 2013; Hussein et al. 2013). A relationship between use of these invasive instruments and colonization of these instruments with resistant organisms and development of infection was emphasized in some studies (Ben-David et al. 2012; Kang et al. 2006; Petrosillo et al. 2013). Use of these instruments rather in patients with a severe clinical picture who require care at ICU may also contribute to high mortality rate.

In our study, mortality was significantly high in patients with colostomy. In the reviewed literature there was only one study investigating effect of presence of colostomy on mortality and no significant association was detected (Michalopoulos et al. 2011). The studies were rather focused on the effect of presence of colostomy/PEG on antibiotic resistance. Researchers have concluded that presence of colostomy/PEG didn’t cause any change in resistance rates (Liu et al. 2012; Chang et al. 2011; Falagas et al. 2006). Further studies focused on relationship of this entity and mortality is needed.

In our study there was no significant relationship between type of surgical procedure and mortality. In a study, having a surgical operation within the last one month wasn’t found to be significantly related with mortality but the type of surgery wasn’t reported (Capone et al. 2013). It’s assumed that pre-op assessment for co-morbid diseases before elective surgical procedures and postponing or cancelling surgical operations in uncontrolled diseases may contribute to lack of high mortality. Also, shorter pre-op and post-op hospital stay may affect natural flora less and this may lower the risk for colonization with resistant microorganisms and risk of infection.

Our findings have shown that, according to EUCAST breakpoint values colistin resistance doesn’t exist in our hospital; however, CR rates was high (43.2%) in accordance with the literature (Alicino et al. 2015; Vardakas et al. 2015; Chen et al. 2012; van Duin et al. 2014; Ulu et al. 2015; Hussein et al. 2013). In a study investigated instrument-related infections developing during 2003–2012 in our country and including all ICUs of 29 hospitals from 10 cities CRKp resistance rate at CLABSI was 6.3% (Leblebicioglu et al. 2014). In two separate studies investigating HAI developing in ICUs during 2009–2011 and 2012 CRKp rate was 22 and 48% respectively (Ulu et al. 2015; Budak et al. 2014). Increasing CRKp incidence over the years are clearly seen in these studies and our CRKp rate is high in line with the data of Candevir et al. which is the closest study to present-time (Ulu et al. 2015). In line with the literature, high CRKp incidence which is a threat in most countries has also grown in our country to a dimension that is posing threat. New studies revealing more clear data and including various regions are needed about this issue.

Contrary to our expectations, there was no significant relationship between appropriate use of empirical antibiotic therapy and survival. Presence of many adverse factors such as co-morbid diseases or negative clinical conditions adversely effecting survival may cancel the positive effects of appropriate use of empirical antibiotic therapy in lowering mortality rates. In similar studies it was reported that conformity of initial therapy with antibiogram test doesn’t lead to a change in survival rate of patients (Ben-David et al. 2012; Pau et al. 2015; Correa et al. 2013; Qureshi et al. 2012).

Our study has some limitations such as its retrospective design which doesn’t permit desired modifications in the design of the study. The antibiotic susceptibility tests were only performed with automated system because microbiology laboratory of our university had a work overload and the personnel were few. Procalcitonin and lactate test results were available only for few cases so that we didn’t mentioned about them in the paper. Since *K. pneumoniae* have different resistance profiles we used many different antibiotics or combinations as treatment. The data about the treatment was too excessive so we just mentioned about empirical therapy.

## Conclusions

Our study has shown that mortality is high in the presence of post-NKp BSI sepsis, septic shock, ICU treatment, meropenem resistance, NKp BSI secondary to pneumonia, kidney failure as the reason for hospitalization and having renal failure as co-morbid disease. Furthermore, mortality was also high in those requiring MV and hemodialysis as supportive therapy, and in presence of UC, CVC and colostomy. On the other hand, in patients who were admitted to the hospital for neurological disorders, PBT diseases, in those whom PBT infections is considered as the source of BSI and in patients who have undergone ERCP mortality rate was lower. In situations leading to an increase in mortality rates rigorous attention is needed in following the rules for infection control measures, wide-spectrum antibiotics should be preferred in empirical therapy and remedial strategies for future should be adopted.

## Abbreviations

NKpBSI: nosocomial *K. pneumoniae* blood stream infection
UC: urinary catheter
PBT: pancreaticobiliary tract
HAI: health care associated infections
CT: chemotherapy
TPN: total parenteral nutrition
HD: hemodialysis
BSI: bloodstream infection
ICU: intensive care units
HR: hazard ratio
CI: confidence interval
SSI: surgical site infection
UTI: urinary tract infection
STI: soft tissue infection
CLABSI: central line-associated bloodstream infection
CVC: central venous catheter
ERCP: endoscopic retrograde cholangiopancreatography
CpR: carbapenem resistance
CpS: carbapenem susceptible
CLSI: Clinical and Laboratory Standards Institute
EUCAST: The European Committee on Antimicrobial Susceptibility Testing
ARF: acute renal failure
CRF: chronic renal failure
GIS: gastrointestinal system
CNS: central nervous system
GUS: genitourinary system
CVS: cardiovascular surgery
TGC: tigecycline
TMPSXT: trimethoprim-sulfamethoxazole
MEM: meropenem
GM: gentamicin
AK: amikacin
LEV: levofloxacin
CIP: ciprofloxacin
TE: tetracyclin
TZP: piperacillin-tazobactam
CES: cefoperazone-sulbactam
CAZ: ceftazidim
FEP: cefepime
SAM: sulbactam-ampicillin
CS: colistin
FDA: Food and Drug Administration
CRKp: carbapenem resistant *K. pneumoniae*
